# Parental Longevity Is Associated With Brain Volumes in Selected Areas

**DOI:** 10.1093/geroni/igaa057.1695

**Published:** 2020-12-16

**Authors:** Qu Tian, David Melzer, Luke Pilling, Luigi Ferrucci

**Affiliations:** 1 National Institute on Aging, Bethesda, Maryland, United States; 2 University of Exeter, Exeter, United Kingdom

## Abstract

A few studies report that parental longevity is associated with faster gait speed, less memory decline, a lower risk of Alzheimer’s disease, and lower white matter hyperintensities. Data on structural neuroimaging correlates of parental longevity and its spatial distribution are limited. This study aims to examine relationships of parental longevity with regional brain volumes. We identified 10,513 participants from UK Biobank (mean age=58±6, ranged40-70, 50%women) with data on parental longevity and information on MRI regional brain volumes that have been related to executive function (dorsolateral prefrontal cortex), memory (hippocampus, parahippocampal gyrus, inferior temporal lobe, middle temporal lobe) and motor function (precentral gyrus, putamen, caudate, corpus callosum). Participants were categorized based on whether at least one parent lived to age 85 or older or neither parent survived to age 85. Associations of parental longevity with each brain volume measure were examined using linear regression, adjusted for age, sex, education, ApoE e4 status, total gray matter atrophy, white matter hyperintensities, hypertension, and glucose. Compared to participants whose both parents died before 85, those with at least one parent surviving to 85 had greater brain volumes in hippocampus, parahippocampal gyrus, inferior temporal lobe, middle temporal lobe, and precentral gyrus in fully adjusted models (Bonferroni corrected p<0.01). There were no significant associations with volumes in dorsolateral prefrontal cortex, putamen or caudate. Parental longevity is associated with preserved brain structure localized in memory- and motor-related cortical regions. These findings support previous reports that parental longevity is associated with better memory and gait with aging.

